# Type 1 diabetes is associated with an increased risk of venous thromboembolism: A retrospective population-based cohort study

**DOI:** 10.1371/journal.pone.0226997

**Published:** 2020-01-14

**Authors:** Yi-Hao Peng, Yu-Sheng Lin, Chia-Hung Chen, Kun-Yuan Tsai, Yi-Chih Hung, Hsuan-Ju Chen, Wei-Chih Liao, Wen-Chao Ho

**Affiliations:** 1 Department of Public Health, China Medical University, Taichung, Taiwan; 2 Department of Respiratory Therapy, Asia University Hospital, Asia University, Taichung, Taiwan; 3 Department of Respiratory Therapy, China Medical University, Taichung, Taiwan; 4 Department of Chest Medicine, Division of Internal Medicine, Asia University Hospital, Asia University, Taichung, Taiwan; 5 Division of Pulmonary and Critical Care Medicine, Department of Internal Medicine, China Medical University Hospital, Taichung, Taiwan; 6 Division of Endocrinology and Metabolism, Chung-Hsin Clinic, New Taipei, Taiwan; 7 Division of Endocrinology and Metabolism, Department of Medicine, China Medical University Hospital, China Medical University, Taichung, Taiwan; 8 Graduate Institute of Clinical Medical Science, China Medical University, Taichung, Taiwan; 9 Management Office for Health Data, China Medical University Hospital, Taichung, Taiwan; 10 Department of Nursing, Asia University, Taichung, Taiwan; Universiti Sains Malaysia, MALAYSIA

## Abstract

**Background:**

It has been unclear whether diabetes mellitus (DM) is positively associated with a risk of venous thromboembolism (VTE). In addition, whether the risk of VTE is altered in patients with type 1 diabetes (T1DM) has rarely been explored.

**Aim:**

We investigated whether patients with T1DM are at a relatively high risk of VTE development.

**Methods:**

We retrieved data from the National Health Insurance Research Database of Taiwan to conduct this retrospective cohort study. The T1DM group consisted of 4967 patients diagnosed as having T1DM before 2003. The non-T1DM group comprised 19 868 age- and sex-matched enrollees without T1DM. Cox proportional hazard regression analysis was used to investigate the hazard ratio of VTE in patients with T1DM relative to those without T1DM.

**Results:**

During a mean follow-up period of 8.61 years, the risk of VTE in the T1DM group was 5.33-fold higher than in the non-T1DM group after adjusting for dyslipidemia, hypertension, stroke, lower leg fracture or surgery, and obesity. Further stratified analysis revealed that the risk of VTE was significantly high in both sexes and in all age groups below the age of 60.

**Conclusion:**

T1DM appears to be an independent risk factor for VTE development.

## Introduction

Diabetes mellitus (DM) was estimated to affect 171 million people globally in 2000 and is projected to rise to 366 million by 2030 [1]. It is a complex metabolic disorder commonly associated with long-term complications of both the macrovascular and microvascular systems [2, 3], and these complications are the leading causes of morbidity and mortality in patients with DM [4, 5]. Although type 1 diabetes (T1DM) only accounts for 5%–10% of all DM, it usually begins earlier in life than type 2 diabetes (T2DM), implying that it may have a more negative effect on a patient’s quality of life and cause a substantial burden to society [6, 7].

Deep vein thrombosis (DVT) and pulmonary embolism (PE), collectively known as venous thromboembolism (VTE), cause considerable morbidity and mortality and are reported as being the most common preventable causes of hospital-related death [8]. Although numerous conditions have been recognized as risk factors for VTE, such as trauma to or surgery on a lower extremity, advanced age, and obesity,[9] it has been estimated that approximately 25%–50% of patients with first-time VTE do not have any readily identifiable risk factors [10].

To date, numerous studies and three meta-analyses on the association between DM and VTE have been reported. Nevertheless, the results of these studies appear to be inconclusive [11–13]. In these analyses, patients with T1DM or T2DM were all included without distinction between each other; whether the risk of VTE is altered solely in patients with T1DM has rarely been explored. Moreover, studies have indicated that the incidence of VTE varies considerably among various ethnic groups, with Asian population appearing to have the lowest incidence compared with Caucasian, Hispanic, African American, or Pacific Islander populations [14, 15]; whether the risk of VTE is altered in Asian patients, such as Taiwanese, with DM, particularly those with T1DM, has also never been explored.

In this study, we investigated whether patients with T1DM have an increased risk of VTE in a retrospective population-based cohort study based on data from the National Health Insurance (NHI) Research Database (NHIRD) of Taiwan.

## Methods

### Data source

The compulsory, universal, single-payer NHI program was established by Taiwan in 1995. It currently covers more than 99% of the population in Taiwan. The NHIRD is derived from the NHI program; this database contains all registry and claims data concerning beneficiaries, including demographic characteristics, disease diagnoses, and dates of outpatient visits as well as hospitalizations, drug prescriptions, examinations, and surgeries submitted for insurance reimbursement. The disease diagnosis was based on the International Classification of Disease, Ninth Edition, Clinical Modification (ICD-9-CM). All data were fully anonymized before we accessed it and the Institutional Review Board of China Medical University Hospital waived the requirement for informed consent (CMUH-104-REC2-115).

### Study cohort and comparison cohort

Patients with and without type 1 diabetes were selected from the NHIRD, and assigned as T1DM and non-T1DM cohort, respectively. The diagnosis of T1DM was highly valid and reliable because for patients applying for reimbursement for T1DM, it is mandatory to have the glucagon stimulation test results and meet the criteria of absence of *β*-cell functional reserve. In addition, any documentation regarding hospitalizations due to ketoacidosis, or results from anti-glutamic acid decarboxylase (anti-GAD) antibody and anti-tyrosine phosphatase-like insulinoma antigen 2 (anti-IA2) were also encouraged to attached if available. Clinical information will be reviewed by medical experts upon reimbursement claim. We identified patients newly diagnosed as having T1DM (ICD-9-CM) before 2003 and defined the date of January 1, 2003 as the index date. Any patient diagnosed as having VTE before the index date was excluded from both cohorts. Finally, 4967 patients with T1DM were enrolled in the study cohort.

### Matching process

For each patient with T1DM in the study cohort, we randomly selected four enrollees in the LHID 2000 without DM and frequency-matched them according to sex, age (per 5-year span), and the year of index date. We excluded any enrollees with VTE before included in both groups. After the matching process, 4967 patients with VTE were included in the T1DM group, 19 868 enrollees without VTE were included in the non T1DM group.

### Covariates

We considered sex, age, dyslipidemia (ICD-9-CM 272), hypertension (ICD-9-CM 401–405), heart failure (ICD-9-CM 428), stroke (ICD-9-CM 430–438), lower leg fracture or surgery (ICD-9-CM 820, 821, 823 or ICD-9 operation code: 81.51–81.54), and obesity (ICD-9-CM 278.xx) as covariates in the analyses.

### Primary outcome

The primary outcome was VTE, including DVT (ICD-9-CM 452 and 453) and PE (ICD-9-CM 415.1; excluding 415.11). Both groups were subject to follow-up from the index date until the first diagnosis of VTE, withdrawal from the NHI, or December 31, 2011.

### Statistical analyses

We summarized the continuous data using mean and standard deviation (SD) and the categorical data using frequency and percentage. The continuous and categorical variables between groups were compared using Student’s t test and the chi-square test, respectively. We calculated the incidence rate of VTE (per 10 000 person-years) as the number of VTE cases divided by the person-time at risk for both the T1DM and non-T1DM groups. Kaplan–Meier analysis was used to estimate the cumulative incidence of VTE in both cohorts. The multivariate Cox proportional hazard regression model was used to measure the hazard ratio (HR) and 95% confidence interval (CI) of VTE-associated risk factors and VTE. The multivariate model was adjusted for sex, age, dyslipidemia, hypertension, heart failure, stroke, lower leg fracture or surgery, and obesity. We further investigated the association between T1DM and VTE in various subgroups according to sex, age, and comorbidity status.

All statistical analyses were conducted using SAS 9.4 statistical software (SAS Institute Inc., NC, USA). Two-tailed p < 0.05 was considered significant.

## Results

**Fig 1:**
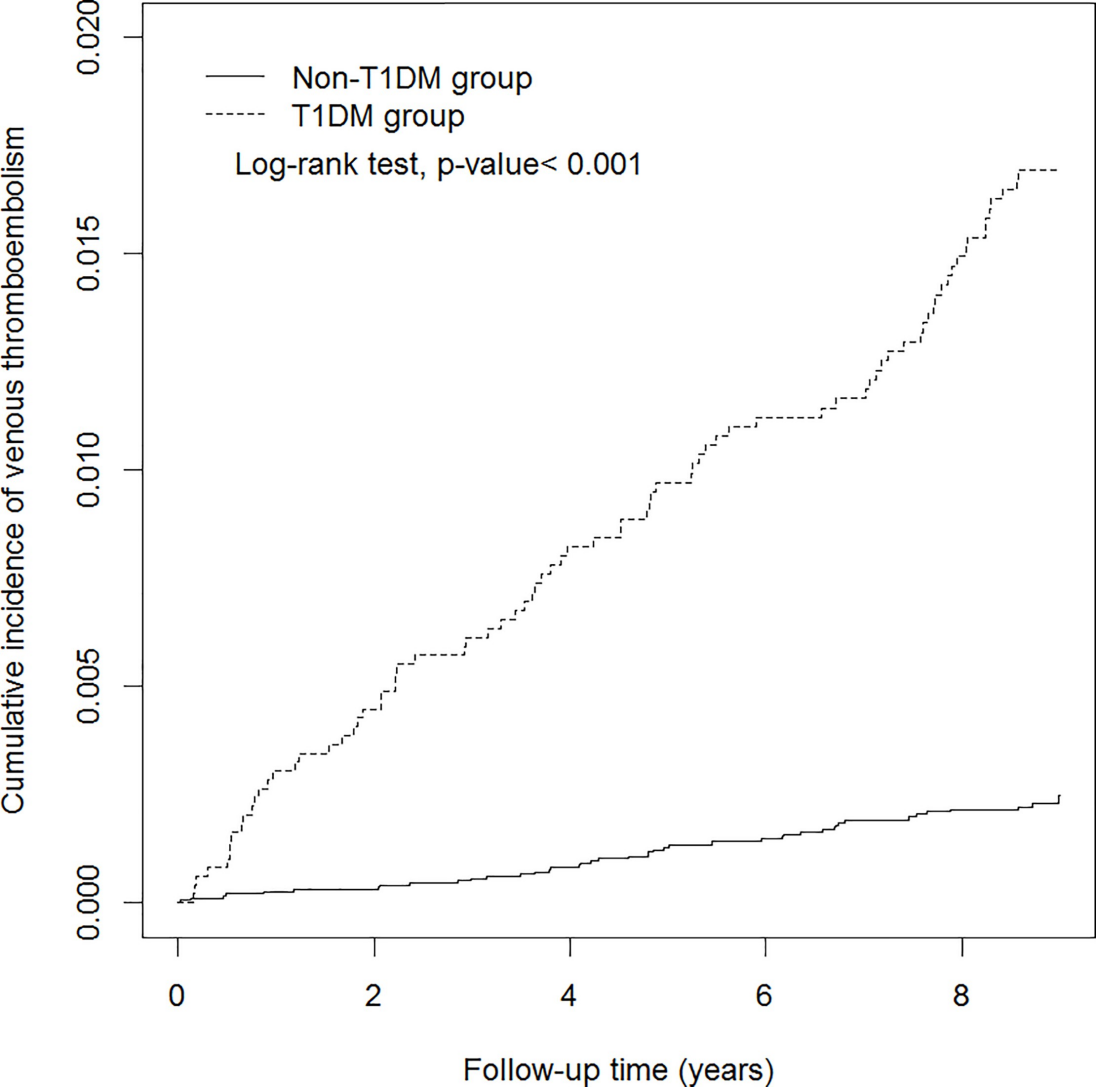
Cumulative incidence curves of venous thromboembolism for groups with and without T1DM.

The characteristics of the demographic factors and comorbidity in both the T1DM and non-T1DM groups are presented in Table 1. The distribution of sex and age did not differ between groups after frequency matching. The mean age was 27 years in both groups (SD = 15 years), and no substantial difference was apparent in the sex distribution (women = 53.55%; men = 46.45%). The T1DM group had a significantly higher prevalence of the comorbidities of dyslipidemia, hypertension, stroke, lower leg fracture or surgery, and obesity than did the non-T1DM group. The cumulative incidence of VTE in both groups is presented in Fig 1. It was significantly higher in the T1DM group than in the non-T1DM group (log-rank test, p < 0.001).

**Table 1:** Baseline demographic factors and comorbidities of study participants according to T1DM status.

	Non-T1DM group N = 19868		T1DM group N = 4967		p-value
Variable	n	%	n	%	
Sex					0.99
Women	10610	53.55	2660	53.55	
Men	9228	46.45	2307	46.45	
Age, years					0.99
< 20	7256	36.52	1814	36.52	
20–39	9104	45.82	2276	45.82	
40–59	2856	14.37	714	14.37	
≥ 60	652	3.28	163	3.28	
Mean (SD)	26.98	(14.66)	26.97	(14.58)	0.96
Comorbidity					
Dyslipidemia	753	3.79	1358	27.34	<0.001
Hypertension	936	4.71	820	16.51	<0.001
Heart failure	79	0.40	77	1.55	<0.001
Stroke	70	0.35	80	1.61	<0.001
Lower leg fracture or surgery	244	1.23	136	2.74	<0.001
Obesity	47	0.24	59	1.19	<0.001

Abbreviation: SD, standard deviation; T1DM, type 1 diabetes mellitus.

During a mean follow-up period of 8.61 years, 45 participants developed VTE in the non-T1DM group and 80 patients developed VTE in the T1DM group, with an incidence of 2.62 and 19.01 per 10 000 person-years, respectively. The Cox proportional hazard regression analysis indicated that the T1DM group had a significantly higher risk of VTE than the non-T1DM group after adjusting for hyperlipidemia, hypertension, stroke, lower leg fracture or surgery, and obesity (adjusted HR = 5.33, 95% CI = 3.57–7.96, Table 2). Neither sex was associated with a higher risk of VTE. Compared with enrollees aged below 20 years, enrollees in the age groups of 20–39, 40–59, and ≥ 60 years all exhibited a significantly higher risk of VTE (Table 2).

**Table 2:** Cox model measured hazard ratios and 95% confidence interval of venous thromboembolism associated with T1DM and covariates.

Variable	Event no.	Person-years	IR	HR (95% CI)	
				Univariate	Multivariate^†^
T1DM					
No	45	171891	2.62	1.00	1.00
Yes	80	42089	19.01	7.28 (5.05–10.49)***	5.33 (3.57–7.96)***
Sex					
Women	68	115331	5.90	1.00	1.00
Men	57	98649	5.78	0.98 (0.70–1.39)	0.94 (0.66–1.34)
Age, years					
< 20	12	79539	1.51	1.00	1.00
20–39	65	98030	6.63	4.40 (2.38–8.14)***	3.49 (1.87–6.54)***
40–59	29	30292	9.57	6.36 (3.24–12.45)***	3.64 (1.78–7.45)***
≥ 60	19	6119	31.05	20.69 (10.04–42.64)***	6.46 (2.78–15.0)***
Comorbidity					
Dyslipidemia					
No	85	196423	4.33	1.00	1.00
Yes	40	17556	22.78	5.27 (3.62–7.67)***	1.08 (0.71–1.65)
Hypertension					
No	71	200124	3.55	1.00	1.00
Yes	54	13856	38.97	11.02 (7.73–15.70)***	3.78 (2.43–5.87)***
Heart failure					
No	118	212985	5.54	1.00	1.00
Yes	7	995	70.38	12.74 (5.94–27.33)***	1.82 (0.81–4.12)
Stroke					
No	119	213024	5.59	1.00	1.00
Yes	6	956	62.76	11.29 (4.97–25.66)***	1.66 (0.68–4.01)
Lower leg fracture or surgery					
No	117	210950	5.55	1.00	1.00
Yes	8	3030	26.40	4.76 (2.33–9.74)***	2.33 (1.13–4.82)*
Obesity					
No	124	213041	5.82	1.00	1.00
Yes	1	938	10.7	1.83 (0.26–13.1)	0.73 (0.10–5.25)

Abbreviations: IR, incidence density rate, per 10,000 person-years; HR, hazard ratio; CI, confidence interval.

† Adjusted for asthma, sex, age (categorical), hyperlipidemia, hypertension, heart failure, stroke, and lower leg fracture or surgery

* p < 0.05

*** p < 0.001.

The multivariate Cox proportional hazard analysis demonstrated that VTE was independently associated with hypertension (HR = 3.75, 95% CI = 2.43–5.87) and lower leg fracture or surgery (HR = 2.33, 95% CI = 1.13–4.82).

Table 3 presents the results of the study population being stratified by sex, age, and comorbidity status. We discovered that both women and men in the T1DM group had a significantly higher risk of VTE than those in the non-T1DM group (adjusted HR = 5.99, 95% CI = 3.43–10.47; adjusted HR = 5.07, 95% CI = 2.85–9.01, respectively). When the study population was stratified by age, we observed that the risk of VTE in the T1DM group was the highest in the age group of 20–39 years (adjusted HR = 16.23, 95% CI = 7.66–34.41) compared with the non-T1DM group and it remained significantly higher in the age groups of < 20 years and 40–59 years (adjusted HR = 4.23, 95% CI = 1.22–14.69; adjusted HR = 3.21, 95% CI = 1.45–7.12, respectively).

**Table 3:** Incidence density rates and hazard ratios of venous thromboembolism according to T1DM status stratified by sex, age, and comorbidity.

	T1DM						Compared to non-T1DM group	
	No			Yes			HR (95% CI)	
Variable	Event no.	Person-years	IR	Event no.	Person-years	IR	Crude	Adjusted ^‡^
Sex								
Women	21	92638	2.27	47	22692	20.71	9.23 (5.52–15.44)***	5.99 (3.43–10.47)***
Men	24	79253	3.03	33	19396	17.01	5.57 (3.29–9.44)***	5.07 (2.85–9.01)***
Age, years								
< 20	5	63482	0.79	7	16056	4.36	5.54 (1.76–17.47)**	4.23 (1.22–14.69)*
20–39	9	78687	1.14	56	19343	28.95	25.62 (12.67–57.80)***	16.23 (7.66–34.41)***
40–59	15	24590	6.10	14	5702	24.55	3.90 (1.88–8.10)***	3.21 (1.45–7.12)**
≥ 60	16	5131	31.18	3	987	30.39	0.99 (0.29–3.42)	0.71 (0.19–2.59)
Comorbidity status ^†^								
No	26	158310	1.64	29	27171	10.67	6.44 (3.79–10.95)***	8.03 (4.69–13.76)***
Yes	19	13581	13.99	51	14918	34.19	2.45 (1.45–4.15)***	3.24 (1.84–5.73)***

Abbreviations: IR, incidence density rate, per 10,000 person-years; HR, hazard ratio; CI, confidence interval.

† Patients with any of the following comorbidities were classified as part of the comorbidity group: hyperlipidemia, hypertension, heart failure, stroke, and lower leg fracture or surgery.

‡ Mutually adjusting for sex, age (continuous), hyperlipidemia, hypertension, heart failure, stroke, and lower leg fracture or surgery.

* p < 0.05

** p < 0.01

*** p < 0.001.

Regardless of an enrollee’s comorbidity status, the patients in the T1DM group had a higher risk of VTE (for patients without comorbidity, HR = 8.03, 95% CI = 4.69–13.76; for patients with comorbidity, HR = 3.24, 95% CI = 1.84–5.73).

## Discussion

In this study, we determined that patients with T1DM were 5.33-fold more likely to develop VTE than those without T1DM; although the prevalence of hypertension and lower leg fracture or surgery was significantly higher in the T1DM group than in the non-T1DM group and was associated with a higher risk of VTE, T1DM still appeared to be an independent risk factor for VTE after adjusting for hypertension, lower leg fracture or surgery, and other comorbidities. A further stratified analysis indicated that the risk of VTE exists in both sexes and appears to be substantially higher in the age group of 20–39 years than in other age groups.

The risk of VTE in patients with T1DM has rarely been explored. Although Stein et al. have investigated the risk of VTE in patients with T1DM by using data retrieved from National Hospital Discharge Survey of US, and observed the relative risk (RR) of VTE in T1DM patients only elevated in patients aged between 40 and 49 years (RR = 1.22 95% CI = 1.21–1.24) [16], they only investigated uncomplicated T1DM patients who did not have obesity, stroke, heart failure and cancer. Moreover, there were insufficient data to calculate the relative risk of VTE in patients age between aged 20 and 39 years. According to our review of the relevant literature, no studies have discerned such a prominent effect as the overall HR of 5.33 in our study. In additions, although three meta-analyses have been published to date, the results appeared to be inconclusive [11–13]. Two of the them have reported that the pooled relative risk (RR) or HR of VTE in patients with diabetes were significantly higher than in the population without DM, with an RR of 1.42 (95% CI = 1.12–1.77) and HR of 1.36 (95% CI = 1.11–1.68), respectively [11, 12]. Another meta-analysis, however, concluded that there was no association or only a modestly positive one with an RR of 1.16 (95% CI = 1.01–1.34) [13]. Most studies included in the aforementioned meta-analyses involved both T1DM and T2DM without distinction, making it difficult to investigate whether the risk of VTE was altered solely in T1DM, compared with the general population.

The exact underlying pathophysiology that would explain the association between T1DM and VTE was not explored in this observational study; we reasoned that T1DM leads to the development of VTE through three aspects: vascular endothelial dysfunction, hypercoagulability, and stagnant blood flow (Virchow’s triad). With respect to vascular endothelial dysfunction, Singh et al. observed that endothelial dysfunction occurs in the first decade of T1DM onset [17], and similar results have also been proposed by other researchers [18, 19]. With respect to hypercoagulability, patients with T1DM were reported to have elevated circulating tissue factor procoagulant activity (TF-PCA) and plasma coagulation factor VIIIa, and the mechanism regulating TF-PCA in T1DM might not be the same as in T2DM [20]. Moreover, in patients with T1DM, complement C3 incorporation into clots is reportedly enhanced [21], plasminogen is glycated and leads to reduced plasmin generation and impaired protein activity [22], these result in hypofibrinolysis. These patients even produce a fibrin clot that is more resistant to fibrinolysis when compared to person without T1DM [23].

With respect to stagnant blood flow, it was reported that patients with T1DM may tend to have a sedentary lifestyle [24] and engage in reduced levels of physical activity around the time of diagnosis because the patients themselves or other parties, such as family members or school teachers, fear the occurrence of exercise-induced hypoglycemia [25] or the patient is unable to exercise because of complications.

The incidence of VTE in the non-T1DM group was 2.62 per 10 000 person-years, which was similar to previous studies reporting the incidence of VTE in the Chinese population. However, these studies have also indicated that the incidence of VTE among the Chinese population appears to be the lowest compared with other ethnic populations such as Caucasian, African American, or Hispanic populations [14, 15]. Whether our findings can be generalized to other ethnic populations warrants further research.

We included obesity as a covariate for adjustment because obesity and overweight are reportedly associated with VTE [26, 27]. However, obesity was not associated with an increased risk of VTE in our analysis. The database we used was primarily created for administrative purpose; therefore, the prevalence and incidence of obesity might have been underestimated in both groups. Thus, we included other obesity-related morbidities, hypertension and hyperlipidemia, as comorbidities for adjustment. Hypertension was associated with an increased risk of VTE, which was consistent with previous studies [28].

Young adults aged between 20 and 39 years represented the largest proportion of both groups (45.8%, Table 1); this was also the age group with the highest risk (HR = 16.23) of VTE in the stratified analysis. We reasoned that most patients with T1DM were first diagnosed before the age of 20, and DM-related complications, including VTE, may take years to develop. Therefore, the highest risk of VTE occurs in the age group of 20 to 39 years. As the patient’s age advances beyond 40 years, increasing DM-related complications may confound the effect of T1DM on VTE risk; thus, the effect of T1DM on VTE risk was not as profound as that between the age of 20 and 39.

The strengths of the present study are the large number of patients with T1DM and the mean follow-up period of over 8 years. In addition, adequate confounding factors were included for adjustment in the analyses to prevent bias the results. However, several limitations should be addressed. First, the data we used were retrieved from the deidentified administrative database, the definition of each disease was based on ICD-9-CM codes, and detailed information regarding the lifestyles of enrollees such as cigarette smoking, alcohol consumption, and family history was unavailable. Second, the enrollees’ clinical information such as laboratory data on HbA1c and coagulation profile, pathological findings, and image results were not available in the NHIRD; however, they are necessary for evaluating disease severity. Finally, although we attempted to include adequate covariates for adjustment and designed the study meticulously, potential confounding factors may still exist that biased our results. Nevertheless, given the validity of the database, large sample size, and long follow-up period, we believe that the relationship between T1DM and VTE remains reliable and can be generalized to the general population.

In sum, we determined that patients with T1DM are at a high risk of VTE, especially between the ages of 29 and 40 years. We suggest that clinicians should be aware that T1DM is a potential risk factor for VTE. Future studies investigating the relationship of T1DM and VTE in other ethnic groups and exploring the underlying pathophysiology are advised to confirm our findings.

## References

[1] Wild S, Roglic G, Green A, Sicree R, King H. Global prevalence of diabetes: estimates for the year 2000 and projections for 2030. Diabetes care. 2004;27(5):1047–53. 10.2337/diacare.27.5.1047 . 15111519

[2] Creager MA, Luscher TF, Cosentino F, Beckman JA. Diabetes and vascular disease: pathophysiology, clinical consequences, and medical therapy: Part I. Circulation. 2003;108(12):1527–32. 10.1161/01.CIR.0000091257.27563.32 . 14504252

[3] Luscher TF, Creager MA, Beckman JA, Cosentino F. Diabetes and vascular disease: pathophysiology, clinical consequences, and medical therapy: Part II. Circulation. 2003;108(13):1655–61. 10.1161/01.CIR.0000089189.70578.E2 . 14517152

[4] Diabetes C, Complications Trial Research G, Nathan DM, Genuth S, Lachin J, Cleary P, et al The effect of intensive treatment of diabetes on the development and progression of long-term complications in insulin-dependent diabetes mellitus. N Engl J Med. 1993;329(14):977–86. Epub 1993/09/30. 10.1056/NEJM199309303291401 . 8366922

[5] Epidemiology of Diabetes Interventions and Complications (EDIC). Design, implementation, and preliminary results of a long-term follow-up of the Diabetes Control and Complications Trial cohort. Diabetes Care. 1999;22(1):99–111. Epub 1999/05/20. 10.2337/diacare.22.1.99 10333910 PMC2745938

[6] Ou HT, Yang CY, Wang JD, Hwang JS, Wu JS. Life Expectancy and Lifetime Health Care Expenditures for Type 1 Diabetes: A Nationwide Longitudinal Cohort of Incident Cases Followed for 14 Years. Value in health: the journal of the International Society for Pharmacoeconomics and Outcomes Research. 2016;19(8):976–84. 10.1016/j.jval.2016.05.017 . 27987648

[7] Pompili M, Forte A, Lester D, Erbuto D, Rovedi F, Innamorati M, et al Suicide risk in type 1 diabetes mellitus: A systematic review. Journal of psychosomatic research. 2014;76(5):352–60. 10.1016/j.jpsychores.2014.02.009 . 24745775

[8] Michota FA. Bridging the gap between evidence and practice in venous thromboembolism prophylaxis: the quality improvement process. Journal of general internal medicine. 2007;22(12):1762–70. 10.1007/s11606-007-0369-z 17891516 PMC2219822

[9] Heit JA, Spencer FA, White RH. The epidemiology of venous thromboembolism. Journal of thrombosis and thrombolysis. 2016;41(1):3–14. 10.1007/s11239-015-1311-6 26780736 PMC4715842

[10] White RH. The epidemiology of venous thromboembolism. Circulation. 2003;107(23 Suppl 1):I4–8. 10.1161/01.CIR.0000078468.11849.66 . 12814979

[11] Ageno W, Becattini C, Brighton T, Selby R, Kamphuisen PW. Cardiovascular risk factors and venous thromboembolism: a meta-analysis. Circulation. 2008;117(1):93–102. Epub 2007/12/19. 10.1161/CIRCULATIONAHA.107.709204 . 18086925

[12] Bai J, Ding X, Du X, Zhao X, Wang Z, Ma Z. Diabetes is associated with increased risk of venous thromboembolism: a systematic review and meta-analysis. Thromb Res. 2015;135(1):90–5. Epub 2014/12/02. 10.1016/j.thromres.2014.11.003 . 25434631

[13] Bell EJ, Folsom AR, Lutsey PL, Selvin E, Zakai NA, Cushman M, et al Diabetes mellitus and venous thromboembolism: A systematic review and meta-analysis. Diabetes Res Clin Pract. 2016;111:10–8. Epub 2015/11/28. 10.1016/j.diabres.2015.10.019 26612139 PMC4752919

[14] White RH, Keenan CR. Effects of race and ethnicity on the incidence of venous thromboembolism. Thromb Res. 2009;123 Suppl 4:S11–7. Epub 2009/06/13. 10.1016/S0049-3848(09)70136-7 . 19303496

[15] Liao S, Woulfe T, Hyder S, Merriman E, Simpson D, Chunilal S. Incidence of venous thromboembolism in different ethnic groups: a regional direct comparison study. J Thromb Haemost. 2014;12(2):214–9. Epub 2013/11/29. 10.1111/jth.12464 . 24283769

[16] Stein PD, Goldman J, Matta F, Yaekoub AY. Diabetes mellitus and risk of venous thromboembolism. Am J Med Sci. 2009;337(4):259–64. Epub 2009/04/15. 10.1097/MAJ.0b013e31818bbb8b . 19365171

[17] Singh TP, Groehn H, Kazmers A. Vascular function and carotid intimal-medial thickness in children with insulin-dependent diabetes mellitus. J Am Coll Cardiol. 2003;41(4):661–5. Epub 2003/02/25. 10.1016/s0735-1097(02)02894-2 . 12598080

[18] Dogra G, Rich L, Stanton K, Watts GF. Endothelium-dependent and independent vasodilation studies at normoglycaemia in type I diabetes mellitus with and without microalbuminuria. Diabetologia. 2001;44(5):593–601. Epub 2001/05/31. 10.1007/s001250051665 . 11380077

[19] Jarvisalo MJ, Raitakari M, Toikka JO, Putto-Laurila A, Rontu R, Laine S, et al Endothelial dysfunction and increased arterial intima-media thickness in children with type 1 diabetes. Circulation. 2004;109(14):1750–5. Epub 2004/03/17. 10.1161/01.CIR.0000124725.46165.2C . 15023875

[20] Singh A, Boden G, Homko C, Gunawardana J, Rao AK. Whole-blood tissue factor procoagulant activity is elevated in type 1 diabetes: effects of hyperglycemia and hyperinsulinemia. Diabetes Care. 2012;35(6):1322–7. Epub 2012/03/14. 10.2337/dc11-2114 22410811 PMC3357241

[21] Hess K, Alzahrani SH, Mathai M, Schroeder V, Carter AM, Howell G, et al A novel mechanism for hypofibrinolysis in diabetes: the role of complement C3. Diabetologia. 2012;55(4):1103–13. Epub 2011/09/16. 10.1007/s00125-011-2301-7 . 21918806

[22] Ajjan RA, Gamlen T, Standeven KF, Mughal S, Hess K, Smith KA, et al Diabetes is associated with posttranslational modifications in plasminogen resulting in reduced plasmin generation and enzyme-specific activity. Blood. 2013;122(1):134–42. Epub 2013/05/24. 10.1182/blood-2013-04-494641 . 23699598

[23] Agren A, Jorneskog G, Elgue G, Henriksson P, Wallen H, Wiman B. Increased incorporation of antiplasmin into the fibrin network in patients with type 1 diabetes. Diabetes Care. 2014;37(7):2007–14. Epub 2014/04/25. 10.2337/dc13-1776 . 24760258

[24] Waden J, Forsblom C, Thorn LM, Saraheimo M, Rosengard-Barlund M, Heikkila O, et al Physical activity and diabetes complications in patients with type 1 diabetes: the Finnish Diabetic Nephropathy (FinnDiane) Study. Diabetes Care. 2008;31(2):230–2. Epub 2007/10/26. 10.2337/dc07-1238 . 17959867

[25] Kennedy A, Narendran P, Andrews RC, Daley A, Greenfield SM, Group E. Attitudes and barriers to exercise in adults with a recent diagnosis of type 1 diabetes: a qualitative study of participants in the Exercise for Type 1 Diabetes (EXTOD) study. BMJ Open. 2018;8(1):e017813 Epub 2018/01/27. 10.1136/bmjopen-2017-017813 29371269 PMC5786070

[26] Samama MM. An epidemiologic study of risk factors for deep vein thrombosis in medical outpatients: the Sirius study. Arch Intern Med. 2000;160(22):3415–20. Epub 2000/12/09. 10.1001/archinte.160.22.3415 . 11112234

[27] White RH, Gettner S, Newman JM, Trauner KB, Romano PS. Predictors of rehospitalization for symptomatic venous thromboembolism after total hip arthroplasty. N Engl J Med. 2000;343(24):1758–64. Epub 2000/12/15. 10.1056/NEJM200012143432403 . 11114314

[28] Huang L, Li J, Jiang Y. Association between hypertension and deep vein thrombosis after orthopedic surgery: a meta-analysis. Eur J Med Res. 2016;21:13 Epub 2016/03/24. 10.1186/s40001-016-0207-z 27004410 PMC4802612

